# The Digital Availability of US Departments of Corrections’ Research Policies: Cross-Sectional Analysis

**DOI:** 10.2196/76835

**Published:** 2025-08-20

**Authors:** Jesse Martinez-Kratz, David Manning, Noel Vest, Jason Glenn, Lauren Brinkley-Rubinstein, Alysse Wurcel

**Affiliations:** 1 School of Medicine, Tufts University, 145 Harrison Ave, Boston, MA, 02111, United States, 1 7346044078; 2 School of Arts and Sciences, Tufts University, Boston, MA, United States; 3 Department of Community Health Science, School of Public Health, Boston University, Boston, MA, United States; 4 Department of History and Philosophy of Medicine, University of Kansas Medical Center, Kansas City, KS, United States; 5 Department of Population Health Sciences, Duke University, Durham, NC, United States; 6 Department of Geographic Medicine and Infectious Diseases, Tufts Medical Center, Boston, MA, United States; 7 Section of General Internal Medicine, Boston Medical Center, Boston, MA, United States

**Keywords:** prison, consent, websites, Department of Corrections, incarceration, research policy

## Abstract

This cross-sectional survey of US Departments of Correction websites found significant heterogeneity in the availability and content of prison-based research policy, procedure, and contact information.

## Introduction

Mass incarceration poses a public health crisis in the United States as a major structural determinant of health [1]. Exposure to incarceration heightens risks of chronic conditions, including cardiovascular disease and cancer, while significantly increasing all-cause mortality [2]. Prior unethical research resulted in federal restrictions on research conducted on criminal-legal involved populations [3]. Varied interpretations of federal regulations have led to heterogenous operationalization of research systems, evidenced in varied state Department of Corrections (DOC) policies in state prisons. Ethical research is needed to measure health-related outcomes for those in prisons; however, these varied interpretations of federal regulations create barriers. We evaluated the accessibility of research-related information on publicly available websites of state DOCs.

## Methods

### Overview

We iteratively developed an 8-point rubric for evaluating the digital imprint and internet-accessible completeness of each state’s DOC’s research policies. Two research team members (JM-K and DM) independently located DOC-based websites (June-August 2023). If the state had a website, the research team sought the presence of a DOC-based research web page. States lacking any DOC website or web page were excluded. For states lacking either a DOC website or a DOC-based research web page, researchers searched “[STATE] Research Department of Corrections” to assess for external websites containing information about prison-based research policies and procedures that appeared within 25 search results.

**Figure 1.**
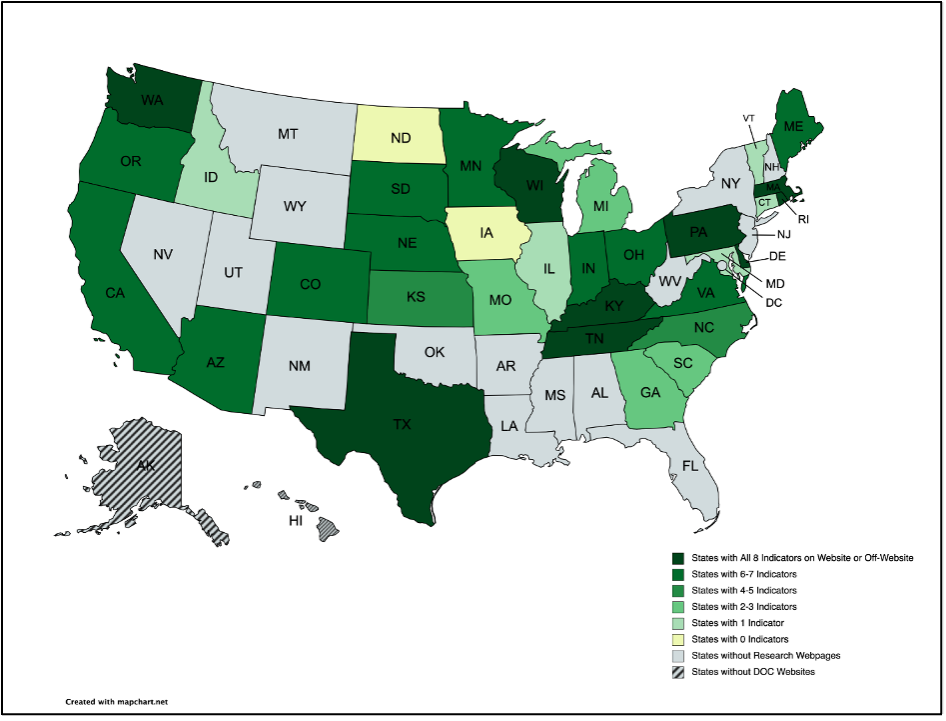
Choropleth map of policy indicators from state DOC websites, generated with MapChart [5]. DOC: Department of Corrections.

If a DOC research website or web page was found for a given state, we assigned up to 8 points, analyzing for the (1) presence of contact information provided for a DOC’s research-related representative and responsiveness within 3 weeks to initiated contact, (2) identification of an institutional review board (IRB) and available policy information. Of available policy information, we evaluated for the presence of policies for (3) staff recruitment, (4) incarcerated people’s recruitment, (5) staff consent, (6) incarcerated people’s consent, (7) staff stipends/payment, and (8) incarcerated people’s stipends/payment. Criteria were piloted on 15 states to ensure interrater reliability (>75%) before assessing the remaining 35, with a final interrater reliability of 94.4%. All data were stored in Qualtrics [4]. Eight-point scores are visualized in Figure 1, and descriptive univariate analysis for categorical variables is reported in Table 1.

**Table 1.** Summary of the availability of websites, availability of policy, contact information, and content of policy information. A web page is referred to as such to delineate it from a website in the same statement.

			States, n/N (%)
Total states			
	States with a DOC^a^-specific^a^ website		48/50 (96)
	Sates with a research web page on the website		33/50 (66)
		If no research web page, research resources found through Google search in the first 25 results	10/50 (20)
		No research web page or easily found information from Google searches	5/50 (10)
Subanalysis of research policies on research web pages			
	Availability of research policies on research web pages		
		Research policies downloadable from web pages	29/33 (88)
		Research policies/procedures directly available on web pages without download	7/33 (21)
		Reference to research policies but not linked to web pages/required separate searches	1/33 (3)
		No reference to policies or procedures	3/33 (9)
		Contact email/phone available	25/33 (76)
	Content of policies on research web pages (available both directly and/or downloadable)		
		Recruitment of staff	22/29 (76)
		Consent of staff	24/29 (83)
		Payment of staff	14/29 (48)
		Recruitment of people who are incarcerated	23/29 (79)
		Consent of people who are incarcerated	24/29 (83)
		Payment of people who are incarcerated	18/29 (62)
		External institutional review board requirements	20/29 (69)

a DOC: Department of Corrections.

### Ethical Considerations

This qualitative study was conducted on publicly available data and thus was deemed nonhuman subject research by the Tufts University Health Sciences IRB.

## Results

Two state DOCs’ (Alaska and Hawaii) websites were unavailable; only 33 of the remaining 48 states’ research web pages were available. Less than half of states had web page–accessible policies available about consent, recruitment, or use of incentives for staff or incarcerated participants (Table 1). We received a response via phone or email from only 23 of the 25 DOC web pages that provided contact information. While similar in number, the states that provided information about incarcerated participants and those that provided information about involved staff only partially overlapped, and several only provided policies for group or the other.

## Discussion

We observed significant heterogeneity in the availability and content of state DOC websites and research web pages, with most DOCs having little information readily available. For instance, only 20 DOC web pages explicitly mentioned IRB requirements, and as few as 14 states had policies available describing staff payments. Barriers to accessing DOC-specific policies have impeded researchers interested in developing prison-based research questions and studies aiming to improve conditions and health outcomes in these settings [6,7]. By extension, the limited accessibility demonstrated herein could hinder future research. Furthermore, limited access to research can be harmful, as demonstrated by HIV’s impact on incarcerated populations [8]. The COVID-19 pandemic reenforced this and displayed how research partnership across carceral, academic, and public sectors are needed to mitigate harm [9,10]. Decreasing barriers and increasing the web-based availability of prison research policies is therefore a reasonable first step to support impactful and feasible research.

Our evaluation was cross-sectional, and websites may change rapidly, reflecting funding or policy, as evidenced by current DOC websites for Alaska and Hawaii that were not available for analysis in 2023. Repeat analysis may reveal changes to other DOC websites as well. Furthermore, public-facing information on prison-based research does not necessarily translate into actual collaboration between researchers and state DOCs, and assessments of virtual information do not capture existing relationships among researchers, prison-based populations, and DOCs. Further analysis should focus on the cascade of research projects (eg, submitted, approved, and published) in each state to better understand how this accessibility translates to research partnerships. Conceivably, increasing the digital availability of DOCs’ research policies will facilitate research initiation and partnerships to improve health outcomes for incarcerated individuals.

## Abbreviations

DOC: Department of Corrections
IRB: institutional review board

## References

[1] Brinkley-Rubinstein L, Cloud DH (2020). Mass incarceration as a social-structural driver of health inequities: a supplement to *AJPH*. Am J Public Health.

[2] Nosrati E, Kang-Brown J, Ash M, McKee M, Marmot M, King LP (2021). Incarceration and mortality in the United States. SSM Popul Health.

[3] Glenn JE (2015). Black Knowledges/Black Struggles: Essays in Critical Epistemology.

[4] (2025). Qualtrics. july 2023 ed.

[5] (2023). MapChart.

[6] Pascoe KJ, Walsh E, Gage BC, Van Son C (2022). Health research in a jail: methodological challenges. Nurs Sci Q.

[7] Neher TL, Udochi AL, Wilson KA, Guillory DM, Zaller ND, Zielinski MJ (2020). Conducting health research in carceral systems: considerations and recommendations. Am J Public Health.

[8] Wurcel AG, Kraus C, Johnson O (2023). Stakeholder-engaged research is necessary across the criminal-legal spectrum. J Clin Transl Sci.

[9] Wang EA, Zenilman J, Brinkley-Rubinstein L (2020). Ethical considerations for COVID-19 vaccine trials in correctional facilities. JAMA.

[10] Saloner B, Kramer C, Song M (2023). COVID-19 restrictions in jails and prisons: perspectives from carceral leaders. Health Aff (Millwood).

